# MOLECULE: Molecular-dynamics and Optimized deep Learning for Entropy-regularized Classification and Uncertainty-aware Ligand Evaluation

**DOI:** 10.1021/acs.jctc.5c01140

**Published:** 2025-09-11

**Authors:** Ivan Cucchi, Elena Frasnetti, Francesco Frigerio, Fabrizio Cinquini, Silvia Pavoni, Luca F. Pavarino, Giorgio Colombo

**Affiliations:** 1 Dipartimento di Matematica “F. Casorati”, 19001 Università di Pavia, Via Ferrata 5, Pavia 27100, Italy; 2 Dipartimento di Chimica, Università di Pavia, Via Taramelli 12, Pavia 27100, Italy; 3 Department of Physical Chemistry, R&D Eni SpA, via Maritano 27, San Donato Milanese (Mi) 20097, Italy; 4 Upstream & Technical Services − TECS/STES − Eni Spa, via Emilia 1, San Donato Milanese (Mi) 20097, Italy

## Abstract

Machine learning (ML) and deep learning (DL) methodologies have significantly advanced drug discovery and design in several aspects. Additionally, the integration of structure-based data has proven to successfully support and improve the models’ predictions. Indeed, we previously demonstrated that combining molecular dynamics (MD)-derived descriptors with ML models allows to effectively classify kinase ligands as allosteric or orthosteric. Extending this approach, we curated a wide and diverse kinase data set (comprising 280 experimentally resolved structures) to train and evaluate a new dual-modal deep neural network classifier, which is tailored to process separately and efficiently the dynamical and structural data to predict the mode of action of a compound. The developed model demonstrated robust classification performance, effective uncertainty handling, and underscored the critical importance of incorporating protein dynamics data. Remarkably, our method maintained high performance even with imputed dynamics data, enabling rapid compound screening and prioritization, without the need for extensive MD simulations.

## Graphical abstract

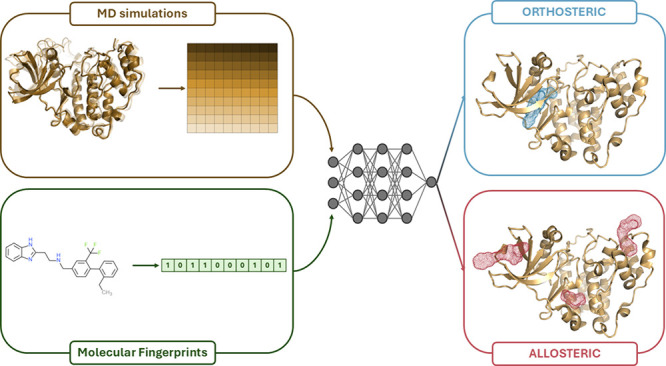

## Introduction

In recent years, Machine Learning (ML) and Deep Learning (DL) methods have been successfully incorporated in drug discovery and drug design campaigns. Some application areas of these models include target identification,^1^ molecule generation,^2^ activity or property prediction^3^ and lead optimization.^4,5^ Overall, using Learning methods to tackle different aspects of the drug discovery pipeline can speed up the selection of candidate leads, reducing experimental workload, costs and risks.

When developing such models, the knowledge about the biological function of the molecules of interest should be proficiently integrated.

One notable example in this framework is the identification of active allosteric ligands to modulate protein activities. Allosteric ligands serve as regulators of protein functions at a distance: they typically engage surfaces that are distal from the active sites of enzymes or from the protein interfaces that underlie functional recognition of partners in the formation of complexes.

In general, they offer an interesting alternative to classical orthosteric competitive inhibitors since allosteric sites are less conserved than active sites among homologous proteins, enhancing the possibilities for selective isoform targeting (Figure 1). Furthermore, allosteric functional modulation can potentially translate into activation/enhancement of the activities of the target, an important point for both fundamental chemical biology studies and applicative drug development. From the fundamental point of view, allosteric gain-of-function probes offer a way to address the sufficiency of an enzyme to drive a particular cellular phenotype and investigate the consequences of overactivation of a certain activity without changing the proteomic profile of the cell with, e.g., induced enzyme overexpression. In terms of drug discovery, activators may be helpful in rescuing a defective activity or in triggering a signaling cascade which may lead to e.g., cell death in the case of cancer. In these cases, it has been reported that the concentration of an allosteric activator may only need to reach an AC10 (activator concentration at 10% maximal activation level) to show a significant phenotypic effect.^6–8^

**Figure 1:**
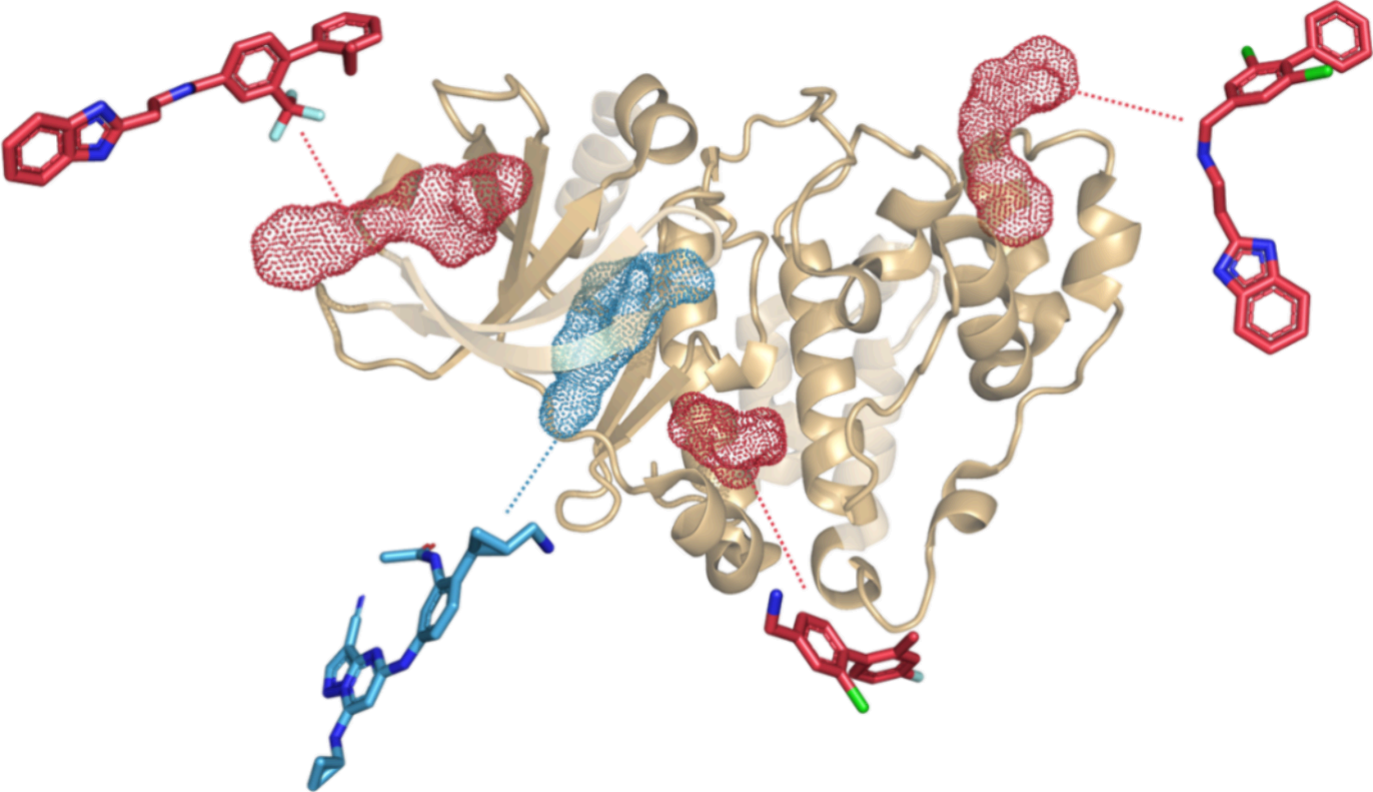
Example of orthosteric (in blue) and allosteric (in red) ligands for Casein kinase 2 alpha 1 and their corresponding binding sites.

From the mechanistic point of view, orthosteric and allosteric ligands show distinct mechanisms of action in determining their impact on protein functions. Importantly, different small molecules may engage different allosteric sites on one single protein, which consequently translates into a variety of regulatory mechanisms and effects on biological pathways and phenotypes. In this framework, while an orthosteric ligand may act simply by displacing the endogenous natural ligand from its binding site, thus shutting down activity, allosteric molecules may act by changing the populations of the structural ensembles of their targets and the mechanisms of interconversion among them. These factors suggest that models able to correctly classify small molecules in terms of their functions as classic inhibitors or allosteric modulators should not neglect the consideration of the impact of ligands on the protein dynamics underlying function.

In our previous work,^9^ we showed that combining Molecular Dynamics derived descriptors and Machine Learning methods could result in the efficient classification of the allosteric vs orthosteric profile of ligands targeting Cyclin Dependent Kinases (CDKs).

Here, we set out to generalize these initial findings, expanding the data set of protein targets to a wide variety of kinase families (covering a total of approximately 280 representative structures for the buildup of the data set), developing a dual-modal entropy-regularized deep neural network classifier, and evaluating the performances of the model and the roles of including dynamics data in defining the allosteric vs orthosteric activity of ligands. In this context, entropy-regularization is used to penalize the exploration of high certainty regions of the data probability distribution, thus producing more diverse outputs and avoiding overfit predictions.

Overall, the evaluation of our model displays a robust classification performance, showing the critical role of including protein dynamics information in the classification task. Importantly, the model retains robust performance and reliable uncertainty handling even when augmented with imputed data.

Imputation, in this context, involves predicting unobserved data from available features. By learning to infer protein dynamics by connecting knowledge about it through simple static descriptors such as ligand fingerprints, the model enables rapid screening of compound libraries and prioritization of candidates with favorable dynamic profiles, without requiring full MD simulations. This supports early stage drug discovery, especially when simulations are impractical or unavailable – e.g., in cases of low-data regimes which are typical of drug selection campaigns, or in cases due to high computational costs when screening large numbers of compounds, lack of knowledge about the ligand’s binding site, etc.

## Materials and Methods

### Data Set Curation

In previous work,^9^ we had prepared a small data set comprising 38 Cyclin-dependent Kinases (CDKs), half bearing an orthosteric ligand and half bearing an allosteric one.

To increase the data set size, we started by accessing the experimental structures with an allosteric ligand, as reported in.^10^ From these complexes, we excluded:

- Structures bearing a ligand already present in the initial CDKs data set.
- Structures bearing a ligand that has been reported to bind in the orthosteric binding site (according to the KLIFS database^11^).

After this filtering step, we were able to collect 133 PDB structures (including the initial 19 CDKs) to include in our extended data set. It should be noted that if a structure contained the same ligand bound in multiple allosteric sites, then multiple entries with each ligand were generated. Therefore, the final number of allosteric entries in our data set was 139 (a 7.3-fold increase in data set size).

Due to the high number of available crystal structures with orthosteric ligands, the filtering step in this case was stricter. In particular, we decided to exclude:

- Mouse proteins.
- Structures bearing a ligand already present in the initial CDKs data set.
- Structures bearing a ligand that has been reported to bind in any of the allosteric binding sites (according to the KLIFS database^11^).
- Structures with missing residues/atoms.

We randomly sampled among the remaining structures until obtaining a final set of 140 entries for the orthosteric class (a 7.4 increase in data set size).

### Preparation of the Protein–Ligand Complexes

Every protein in the data set was prepared using the Protein Preparation Wizard tool in Schrödinger-Maestro suite release 2020–4 (Schrödinger, LLC, New York, NY; Prime, Schrödinger, LLC, New York, NY, 2020, www.schrodinger.com). The preparation steps included removal of original hydrogens, addition of missing chains, assignment of protonation states (at pH = 7 ± 2, using Epik) and optimization of H-bond geometries (at pH = 7, using PROPKA^12^). If the structure had missing residues, they were modeled with Bioluminate’s “Protein linker design” utility in Maestro (Schrödinger, LLC, New York, NY; Prime, Schrödinger, LLC, New York, NY, 2020, www.schrodinger.com) and assigned the correct protonation states. Finally, hydrogens atoms in the protein and the solvent (if present) were removed. For the parametrization of the protein, the AMBER ff14SB force field was used.^13,14^

Ligands’ preparation was carried out using Gaussian 16.^15^ First, an optimization step was performed using the B3LYP hybrid functional for DFT calculation:^16^ the 6–31+g­(2d,p) basis set^17^ was selected and the tight convergence criteria for the SCF cycles was employed. Once the structure was optimized, single point calculations at Hartree–Fock level were performed to obtain the charges according to the Merz–Singh–Kollman scheme.^18^ Generation of parameters was performed using AMBER’s “antechamber” package.^13,14^

Finally, the complex was assigned hydrogens, solvated in a cuboidal box of TIP3P water molecules and neutralized by adding a suitable number of either Cl^–^ or Na^+^ ions. All these steps were carried out using AMBER’s “tleap” tool.^14^ If needed, the structures underwent a minimization step using AMBER’s “parmed” to lower the energy of the structure itself. In this case, the maximum number of minimization cycles was set to 100.

### Molecular Dynamics Simulations and Generation of Descriptors

For each of the complexes, atomistic molecular dynamics (MD) simulations were carried out using the AMBER package (version 20).^13,14^ Four independent replicas were conducted, and each replica consisted of multiple preproduction steps (i.e., minimization, solvent equilibration, heating and equilibration) and a 100 ns of production. For a detailed description of each of the steps, please refer to.^9^

In our previous models,^9^ we manually selected six residues for each structure as representatives of the ATP’s binding site. The chosen residues were involved in the calculations to derive different MD-based descriptors, to describe the motion of the ATP binding site in the presence of an orthosteric or allosteric ligand.

Due to the size of the extended data set, selection of the six residues was performed by first superimposing each structure to one reference complex (PDB code: 1fin) and identifying the closest amino acids with respect to the six residues in the reference (namely Ile10, Lys33, Val64, Glu81, Leu134 and Asp145) with an in-house python script.

Once the residues had been selected, the dynamical descriptors were calculated. In detail:

- Distance between each pair of selected residues. This calculation was performed using the ‘distance’ command in the AMBER cpptraj utility,^19^ which determines the distance between the centers of mass of each residue in the selected mask.
- Root-mean-square deviation (RMSD) of the selected residues. This calculation was performed using the ‘rms’ command in cpptraj. All six residues were included in the same atom mask, and the first frame of the simulation was used as a reference structure.

For both distance and RMSD analyses, the calculation was carried out either considering all the atoms of the six chosen residues (i.e., including the atoms in the side chains) and considering only backbone atoms (i.e., C, CA and N).

Therefore, two sets of data were generated for a single replica, leading to eight (2 sets * 4 MD replicas) samples for a single ligand-protein complex.

Each sample comprises a matrix of shape (16, 2000), where rows represent the dynamical descriptors (i.e., the RMSD and the 15 pairwise distances between the six selected residues), while columns correspond to time frames saved every 0.05 ns over a 100 ns trajectory. Each row is standardized individually using z-score normalization to ensure comparability across descriptors.

Finally, to encode the chemical structure of the ligand, Morgan fingerprints^20^ were computed using RDKit (RDKit: Open-source cheminformatics. https://www.rdkit.org) with the following parameters: radius = 2, length = 2048, chirality = True.

### Model’s Architecture

We propose a deep learning architecture designed to integrate dynamic time-series data with static binary features for a classification task. To address the heterogeneous nature of the inputs, the model processes matrix-shaped (time-series) and vector-shaped (binary) data through two specialized branches. These branches independently extract relevant representations from each modality, which are then fused in a shared decision-making component for final classification. The full model architecture is illustrated in Figure 3.

The choice of a dual-branch architecture is directly motivated by the heterogeneous nature of the input data. For the temporal branch, a 1D Convolutional Neural Network (CNN) was selected due to its proven effectiveness in modeling sequential dependencies in a computationally efficient manner. Through successive convolution and pooling operations, CNNs can automatically learn hierarchical temporal features, capturing both local and global patterns relevant to protein behavior. This makes them particularly well suited for time-series analysis and is consistent with recent advances in molecular simulation studies,^21^ where CNNs have successfully extracted temporal and structural descriptors^22,23^ from MD trajectories. In parallel, a fully connected network was employed for the static binary data, learning a compact and dense representation (embedding) from the high-dimensional features. The representations from both branches are then concatenated, a common and effective fusion strategy that enables the integration of dynamic and static information, providing a more comprehensive basis for classification.

- **Temporal branch**: The first input consists of a temporal signal with 16 features across 2000-time steps. This input is processed by a 1D convolutional neural network (CNN) composed of two convolutional blocks. Each block includes a convolutional layer (with 32 and 64 filters, respectively), followed by batch normalization,^24^ ReLU activation,^25^ and max-pooling. Kernel sizes are set to 5 and 3 with strides of 2, enabling hierarchical temporal feature extraction while progressively reducing sequence length. The output is flattened and passed through a fully connected subnetwork with 16 and 8 units, respectively, each followed by batch normalization, ReLU activation, and dropout (with dropout probability p = 0.7).^26^
- **Binary branch**: The second input is a binary vector of length 2048, representing static chemical features. This input is passed through a parallel fully connected network composed of two layers with 16 and 8 units. Each layer is also followed by batch normalization, ReLU activation, and dropout (p = 0.7), mirroring the architecture of the temporal branch to enable comparable embedding dimensionality.
- **Common branch**: The outputs from both branches (each of dimension 8) are concatenated and fed into a shared classification head. This head comprises a fully connected layer with 8 units followed by batch normalization, ReLU activation, and dropout (p = 0.7). The final output layer is a fully connected layer with 2 units, corresponding to the output logits for binary classification.

*Batch Normalization (BN)* helps stabilize training by normalizing layer activations, reducing internal covariate shift, and enabling faster convergence. It also acts as a regularizer, improving generalization. *Dropout* is used to prevent overfitting by randomly setting a fraction of input units to zero during training. This encourages the model to learn redundant features, enhancing generalization on unseen data. Together, BN and dropout improve model stability, speed up learning, and reduce overfitting, especially given the complexity and heterogeneity of the input data.

To enhance model robustness and encourage confident predictions, we employ a **dynamic focal loss** (*FL*)^27^

$$FL\left(y,ŷ;\gamma \right)=-y{\left(1-ŷ\right)}^{\gamma }\log\left(ŷ\right)-\left(1-y\right){ŷ}^{\gamma }\log\left(1-ŷ\right)$$

where *y* is the true label, *ŷ* represents the predicted probability for a given class, and γ is the focusing parameter. By adjusting the value of γ, the FL prioritizes the harder-to-classify samples, thus the ones that have lower classification probability. To stabilize training in the early stages and progressively emphasize harder (less confident) samples, we gradually increase γ over the first 10 epochs according to the schedule:

γ=min(2.0,0.2·epoch)

This progressive increase helps stabilize training during the initial phases by allowing the model to adapt its focus over time, placing more emphasis on less confident predictions as training progresses. In addition, we incorporate an **entropy-based regularization** term (*H*) to penalize overly uncertain predictions.^28^ The final loss function is given by

L=FL(y,ŷ;γ)+λ·H(ŷ)

where H(ŷ)=−∑ŷlog(ŷ) and λ controls the contribution of the entropy term. This regularization encourages the model to produce more decisive (low entropy) output distributions, complementing the effect of focal loss.

We optimize the model using the **AdaBelief optimizer**,^29^ configured with a learning rate of 10^–3^, ϵ = 10^–16^, β_1_ = 0.9, β_2_ = 0.999, and decoupled weight decay^30^ of 10^–2^. The model is trained for 200 epochs using mini batches of size 32. AdaBelief is chosen for its ability to combine the fast convergence of adaptive optimizers like Adam with the generalization performance of SGD by adapting the step size. A relatively low learning rate (10^–3^) is adopted, as empirical evaluations revealed that higher values led to training instability and overfitting. In our setting, the combination of a high dropout rate (0.7), heterogeneous input modalities, and complex feature interactions likely increased the model’s sensitivity to large parameter updates, thereby necessitating a more conservative learning rate to ensure stable and reliable convergence.

During each iteration, the model performs a forward pass, computes the dynamic focal loss with entropy regularization, backpropagates the gradients, and updates its parameters accordingly. To maintain training stability and control model capacity,^26^ we apply max-norm regularization with a threshold of 3.0 to the weights of all convolutional and fully connected layers. Additionally, we initialize the model parameters to promote effective optimization: Kaiming normal initialization^31^ is used for all linear layers to match the ReLU activation, and biases are set to zero; for convolutional layers, we also apply Kaiming normal initialization to encourage stable gradient flow early in training. The training dynamics are illustrated in Figure S1 (Supporting Information), which shows the evolution of training and validation loss across epochs.

### Feature Importance Analysis

To investigate the contribution of the two input branches (dynamical matrix input and fingerprint vector input) to the model’s predictions, we performed feature importance analysis using Integrated Gradients (IG).^32^ Since our model processes two distinct inputs, we introduced a joint wrapper that merges both branches and enables compatibility with IG, following similar adaptations used in multimodal attribution studies.^33^ Feature attributions were computed separately for the matrix and vector inputs and then normalized to obtain relative importance scores. The results are presented in terms of:

- Total Attribution: the sum of absolute attributions across all features within each input type, expressed as a percentage of the total attribution from both branches. This metric captures the overall contribution of each input modality to the model’s prediction, providing a global view of input relevance.^34^
- Mean Attribution: the percentage contribution based on the mean absolute attributions. This metric reflects how much on average, individual features within each input branch contribute to the model’s prediction.

This analysis allows the quantification of the relative influence of the dynamical and fingerprint features on the model’s decision-making process.

### Imputation Strategy

Since binary fingerprint descriptors are more easily accessible than dynamical simulation data, we developed an imputation strategy to reconstruct the eventually missing dynamical component. Specifically, we trained an auxiliary model, referred to as the *imputer*, which learns to predict the key features of the dynamical data, an 8-dimensional vector, using only the binary fingerprints as input. This vector corresponds to the output of the fully connected subnetwork in the original temporal branch. The imputer is implemented as a fully connected neural network that maps the 2048-dimensional binary input to this 8-dimensional embedding space. It is trained using the mean squared error (MSE) loss function for 200 epochs with the Adam optimizer,^35^ with a learning rate of 10^–5^, and weight decay of 10^–3^ (see Figure 7for an overview of the imputer-augmented architecture).

During inference, the imputed latent vector is concatenated with the output of the binary branch and passed through the shared classification head, allowing the model to maintain its full predictive capacity while relying solely on the more readily available binary features.

### Evaluation Protocol

To better reflect the model’s confidence and mitigate the risk of uncertain predictions, we adopted a threshold-based decision strategy. The model outputs raw logits, which are first transformed into class probabilities using the softmax function.^36^ If the highest predicted class probability for a given input did not exceed this threshold, the sample was assigned to a third ″uncertain″ class. This approach enabled a clear distinction between confident classifications and ambiguous cases, thereby enhancing interpretability and model reliability.

Following this strategy, we computed standard classification metrics (accuracy and F1-score)^37^ based only on the confidently classified samples. To further assess agreement beyond chance, we included Cohen’s kappa score,^38^ which measures the agreement between the predicted and true label. Finally, we visualized the classification results using a confusion matrix.

## Results

### Data Set Structure

To create a suitable data set for our classification purposes, we created a new curated ensemble of approximately 280 experimentally resolved ligand-protein complexes, which includes 73 different protein kinases.

Each of these structures underwent short (i.e., 4 replicas of 100 ns each) Molecular Dynamics (MD) simulations, to generate descriptors that encode the motion of the ATP binding site in the presence of an orthosteric or allosteric compound. In detail, these descriptors are pairwise distance and root-mean-square deviation of six reference amino acids, collected in a matrix of shape (16, 2000). These descriptors were calculated at every time step of a MD replica and unlike our previous approach,^9^ where some representative time frames were selected for each descriptor to reduce dimensionality, we decided to retain the complete time series for all descriptors. This choice is motivated by the need to preserve the full temporal resolution of molecular fluctuations, which may encode subtle but critical signals related to allosteric or orthosteric behavior. Prior studies^39,40^ have demonstrated that discarding temporal granularity can result in the loss of important dynamic features, particularly in systems with complex or time-lagged responses to perturbations.

Additionally, the calculations were performed considering both all the atoms and only the backbone atoms of the residues. Therefore, we obtained eight (4 replicas * 2 sets of atoms considered) set of data for each complex.

To ensure descriptor comparability and minimize scale-induced biases during model training, each of the 16 time series is independently normalized via z-score standardization.^41,42^

Finally, we generated a 2048-bit Morgan Fingerprint vector to encode the chemical structure of the ligand in the complex.

The data set was subsequently divided into training, validation, and test sets using an 80:10:10 ratio. The split was performed at the level of the protein–ligand complex, ensuring that all samples derived from a given structure are assigned to a single subset. In this way we eliminate any potential data leakage, thereby preserving the integrity of model evaluation. This is particularly important in the context of conformational ensemble learning, where structural similarity between replicas could otherwise lead to inflated performance estimates.

Before proceeding with the training of the classifier, we investigated the distribution of the samples in our data set. For this purpose, we visualized the UMAP (Uniform Manifold Approximation and Projection^43^) projection of data points. In detail, the 2-dimensional embedding was constructed by setting the number of neighbors to 20, selecting Jaccard distance as the metric and setting the number of runs to 1000 epochs.

As illustrated in Figure 2, the data set does not exhibit a clear separation between the two classes, underlining the complexity of the classification task.

**Figure 2:**
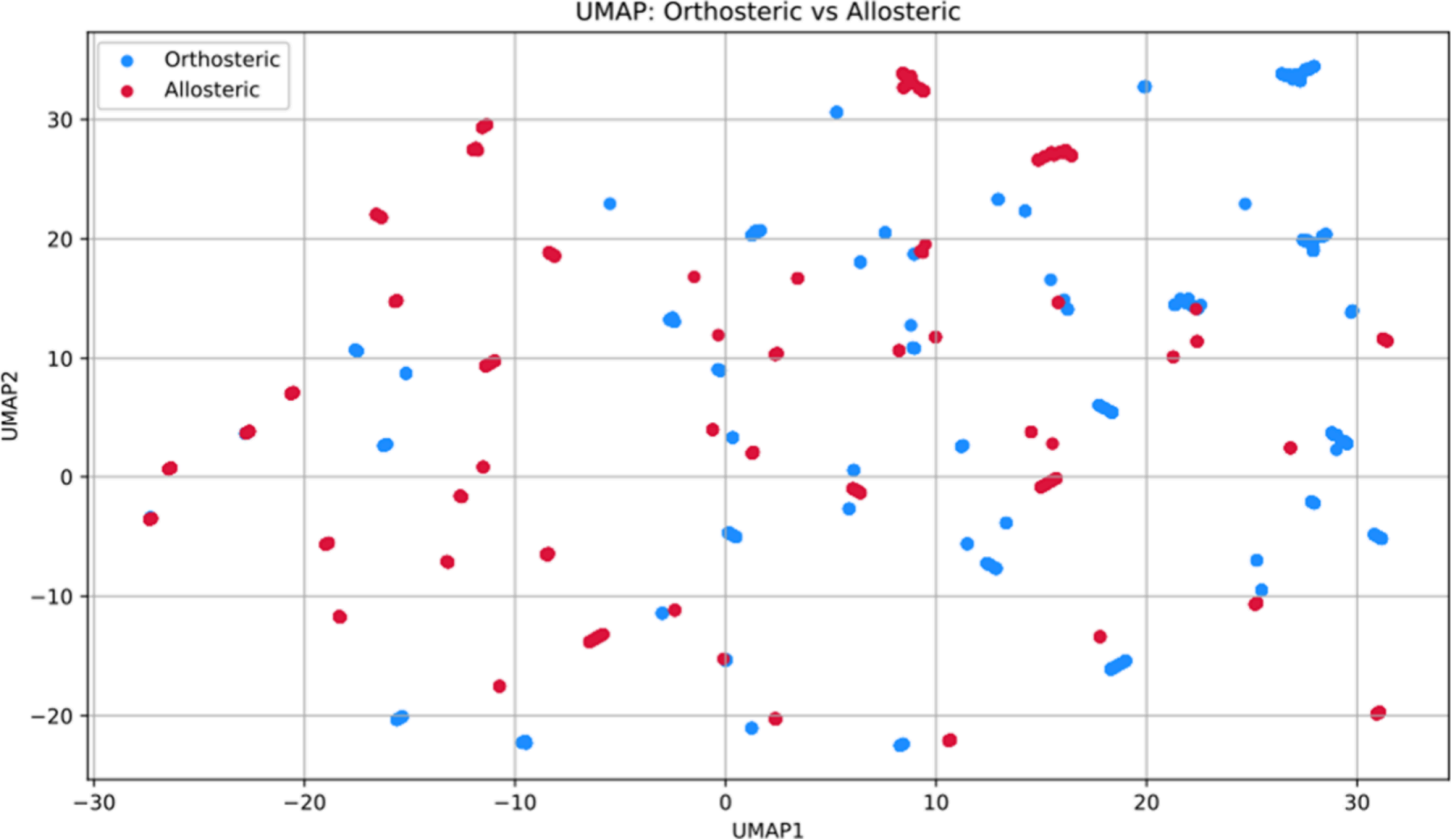
UMAP projection of the data set highlighting orthosteric (in blue) and allosteric (in red) classes.

For this reason, we decided to move away from classic Machine Learning models that we previously used for classification in a much smaller and restricted ensemble of targets and opted for a more complex Deep Learning architecture.

### Overview of the Classifier Architecture

The final data set integrates different types of descriptors (structural vs dynamical), encoded in different forms (binary vector vs matrix of time series).

The proposed architecture (depicted in Figure 3) reflects a design choice to handle the multimodal nature of the data set. The dual-branch structure enables the model to learn modality-specific representations before merging them into a unified latent space for decision making. This separation is critical, as it allows each branch to extract tailored features: convolutional layers in the temporal branch capture temporal dependencies and local patterns across time, while fully connected layers in the binary branch effectively distill the sparse, high-dimensional fingerprint vectors.

**Figure 3:**
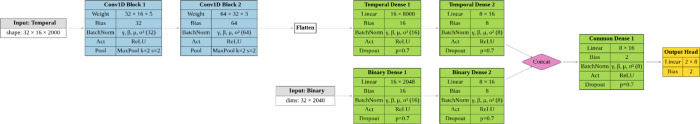
Schematic representation of the proposed deep learning model. The architecture integrates two distinct input modalities: a temporal input (top branch), processed through a two-block 1D convolutional neural network followed by fully connected layers, and a binary input (bottom branch), processed through a parallel fully connected subnetwork. The outputs from both branches are concatenated and passed through a shared classification head to produce the final prediction.

To enhance generalization and mitigate overfitting – particularly important given the relatively small data set size and the high-dimensional input space – we employed batch normalization, dropout, and max-norm regularization. We deliberately used a relatively high dropout rate (0.7) to enforce strong regularization. This choice was motivated by the complexity and heterogeneity of the input features, where smaller dropout rates (e.g., 0.3 and 0.5) were empirically tested but failed to prevent overfitting, leading to unstable generalization. In contrast, the higher rate encouraged the network to develop more robust and redundant representations, thereby improving its ability to generalize. This strategy is also supported in the literature: dropout is consistently regarded as one of the most effective defenses against overfitting in deep networks, and its regularizing power persists even under limited data conditions.^44^ Importantly, training remained stable (Figure S1) thanks to the use of AdaBelief optimizer, whose robustness to noise and adaptive learning behavior ensured convergence even under strong regularization.^45,46^

To better reflect the model’s confidence and mitigate the risk of ambiguous predictions, we adopted a threshold-based decision strategy: after computing class probabilities via the softmax function, samples with a maximum predicted probability below a predefined threshold were assigned to a third ″uncertain″ class.

### Model’s Performance Based on Confidence Threshold

We evaluated the performance of our classifier by examining the impact of different confidence threshold values on the assignment of predictions to the uncertain class (see Table 1).

**Table 1:** Classification Performance under Different Confidence Thresholds on Training and Test Sets

	threshold = 0.5		threshold = 0.8		threshold = 0.95	
metric	train	test	train	test	train	test
accuracy	100.00%	92.86%	99.94%	85.71%	98.88%	69.64%
F1 score	100.00%	92.86%	99.97%	89.73%	99.43%	80.17%
Cohen’s Kappa	1.00	0.86	1.00	0.74	0.98	0.52

At a standard threshold of 0.5, the model achieved its best performance on the test set, with an accuracy and F1-score all reaching 92.86%, and a Cohen’s kappa of 0.86, indicating strong agreement beyond chance. The test confusion matrix shows strong generalization performance, with both orthosteric and allosteric classes predicted with 92.86% accuracy. Misclassifications are evenly distributed across the two main classes, and all samples are confidently classified (see Figure 4).

**Figure 4:**
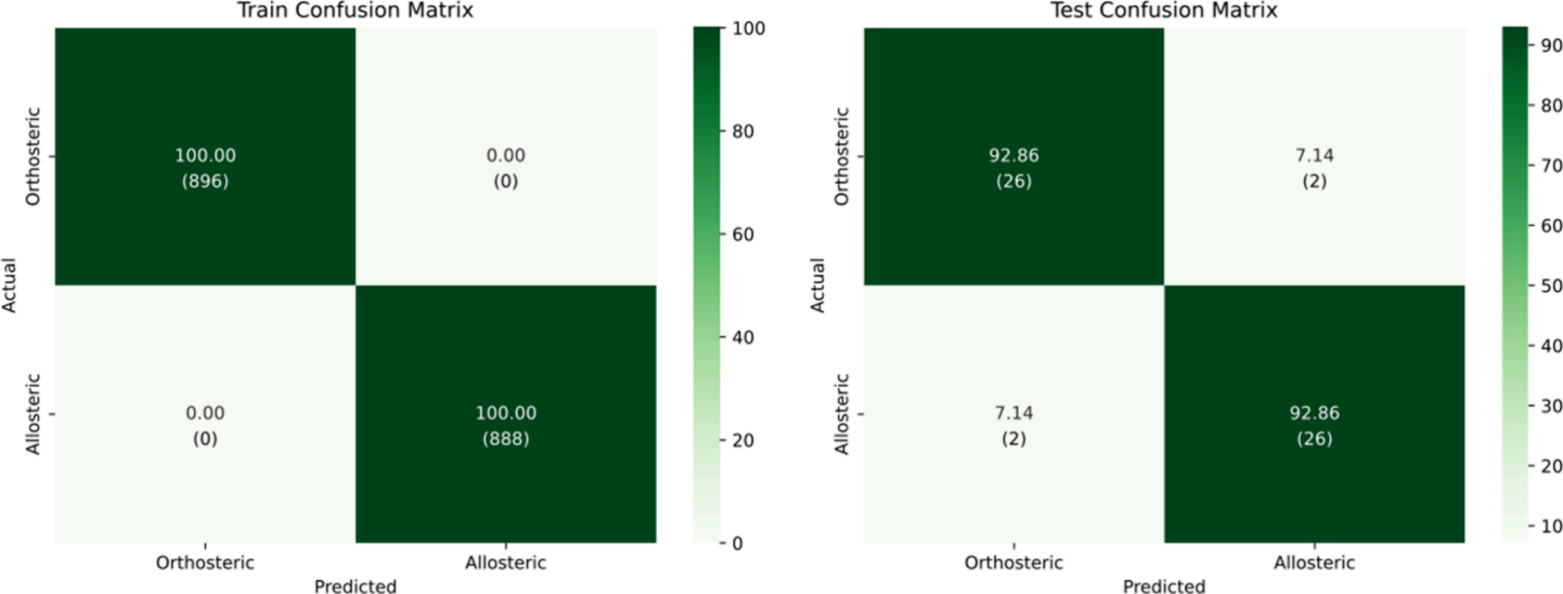
Confusion matrices for train and test sets at confidence threshold = 0.5.

When increasing the threshold to 0.8, the model became more selective in its predictions. This led to a slight drop in test F1-score (89.73%), and Cohen’s kappa dropped to 0.74. This suggests the model maintained high confidence when making predictions, at the cost of increased uncertainty. As shown in Figure 5, the model becomes more selective.

**Figure 5:**
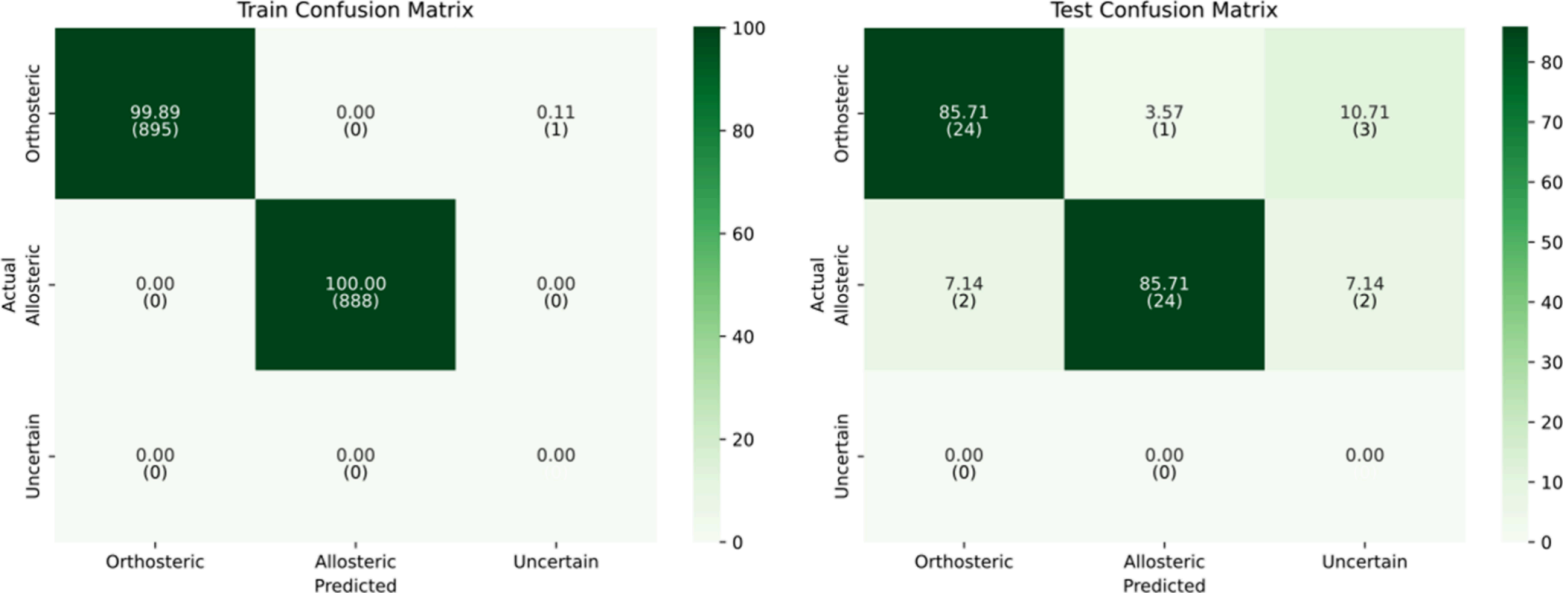
Confusion matrices for train and test sets at confidence threshold = 0.8.

In the train set, the classification remains near perfect, with a single orthosteric sample falling below the threshold and being assigned to the uncertain class. The test set shows balanced performance across classes (85.71% for both), but now includes a few samples assigned to the uncertain class – 3 orthosteric and 2 allosteric. Importantly, these uncertain classifications help filter ambiguous cases without introducing false confident predictions, illustrating the effectiveness of the uncertainty-aware decision strategy.

Finally, at the highest threshold of 0.95, model performance decreased, with test accuracy at 69.64%, F1-score at 80.17%, and Cohen’s kappa at 0.52. This reflects the model’s increased conservativeness under stricter confidence criteria. Figure 6illustrates the impact of this threshold: on the training set, the model maintains strong performance, with only 2.23% of orthosteric samples labeled as uncertain. On the test set, the model classifies 11 orthosteric and 4 allosteric samples as uncertain, indicating a more cautious decision process. Although this results in a lower overall accuracy, the model focuses on high-confidence outputs and avoids making predictions when certainty is low.

**Figure 6:**
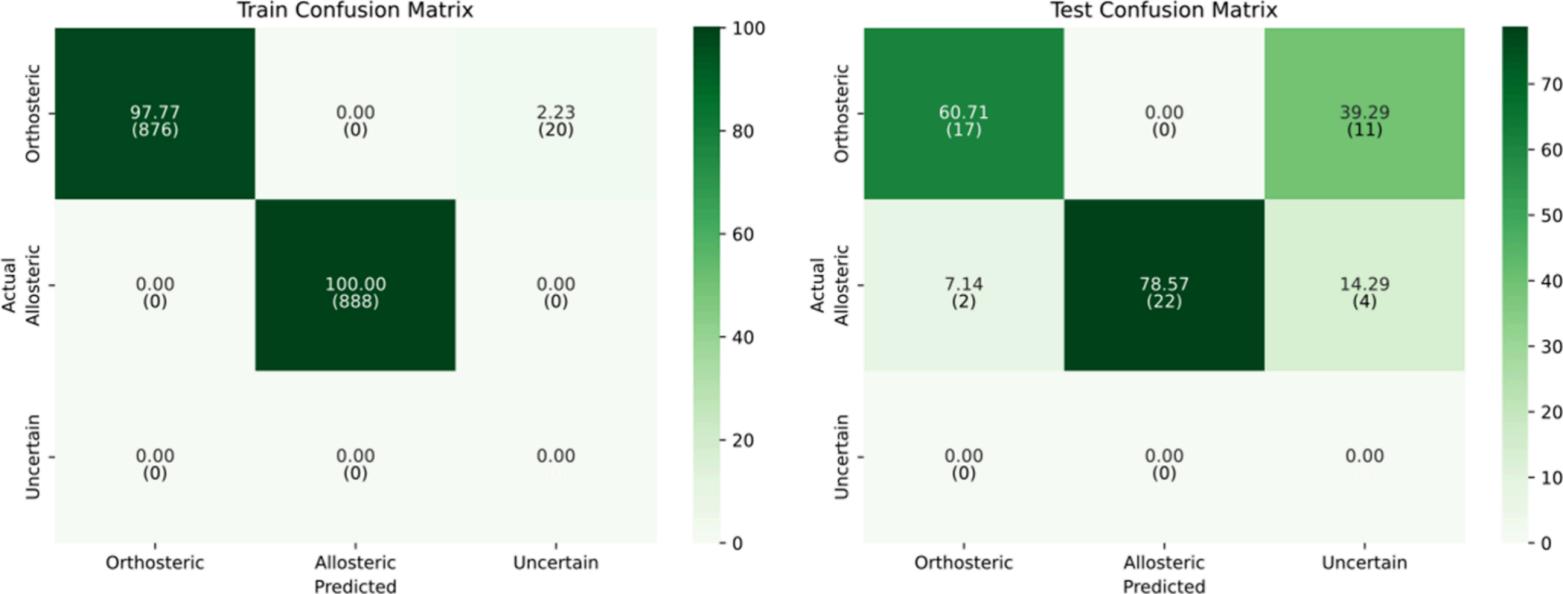
Confusion matrices for train and test sets at confidence threshold = 0.95.

These results highlight a trade-off between model confidence and classification coverage. While raising the threshold increases the number of uncertain classifications, it allows the model to focus on more confident predictions. Notably, at a threshold of 0.8, the model maintains very good performance, and this suggests that even with a moderate increase in uncertainty, the model can provide highly reliable predictions. The detailed classification outputs for each ligand are provided in the Supporting Information (Tables S2–S4).

### Evaluation of Feature Importance

To validate the use of both dynamical and structural descriptors for training of the model, we performed an Integrated Gradients^47^ attribution analysis (please refer to Material and Methods for details on the method). As described in Table 2, the results suggest that the dynamical matrix input plays a major role in guiding the model’s predictions. It accounts for over 90% of the total attributions across both classes, indicating a stronger overall contribution compared to the fingerprint input. However, when examining the average attribution per feature, the fingerprint input shows higher relative values (∼62%). This difference may be attributed to the higher dimensionality of the dynamical input, which spreads its importance across many more features. Consequently, if the dimensionality of the dynamical representation were reduced – perhaps by retaining only the most informative components – its average per-feature attribution might increase. These findings highlight both the overall importance of the dynamical features and the potential for improving their interpretability through dimensionality reduction or feature refinement.

**Table 2:** Distribution of Predictive Contribution between Input Modalities across Sample Types

type	orthosteric	allosteric	average
Tot dynamics	90.45%	90.62%	90.53%
Tot fingerprints	9.55%	9.38%	9.47%
Avg dynamics	37.73%	38.21%	37.97%
Avg fingerprints	62.27%	61.79%	62.03%

### Imputation Strategy and Evaluation

Although the feature analysis results suggest that the dynamical descriptors play a fundamental role in the outcome of the classification task, it is evident that binary fingerprint descriptors, such as those generated using the Morgan algorithm, are significantly more accessible than MD-derived features. Indeed, running MD simulations – especially in early stages of drug discovery – might be impractical due to high cost in terms of computational resources and time. Furthermore, one might not know a priori information on the binding site of the ligand. This is especially critical in the case of proteins for which multiple allosteric sites have been identified, such as kinases.^10^ However, to directly assess the contribution of the binary descriptors, we trained a binary-only version of the model. This baseline shares the same architecture as the complete multimodal network, with the sole difference that the dynamical branch is omitted and the common branch receives input only from the binary pathway. As reported in the Supporting Information (Table S1), this binary-only model displays substantially weaker predictive performance and poor robustness under stricter thresholds, confirming that the binary input alone is insufficient for the classification task.

Therefore, we introduced an imputer mechanism to reconstruct the latent representation of the temporal branch directly from static ligand features. The imputer consists of a fully connected neural network trained to map the 2048-bit binary input to an 8-dimensional embedding that mimics the output of the convolutional and dense layers of the temporal branch. By minimizing the mean squared error between predicted and true embeddings, the imputer learns to approximate the dynamical encoding based solely on chemical fingerprint data. During inference, the imputed embedding is concatenated with the output of the binary branch and forwarded to the shared classification head, enabling the model to retain predictive capacity even in the absence of time-resolved inputs.

**Figure 7:**
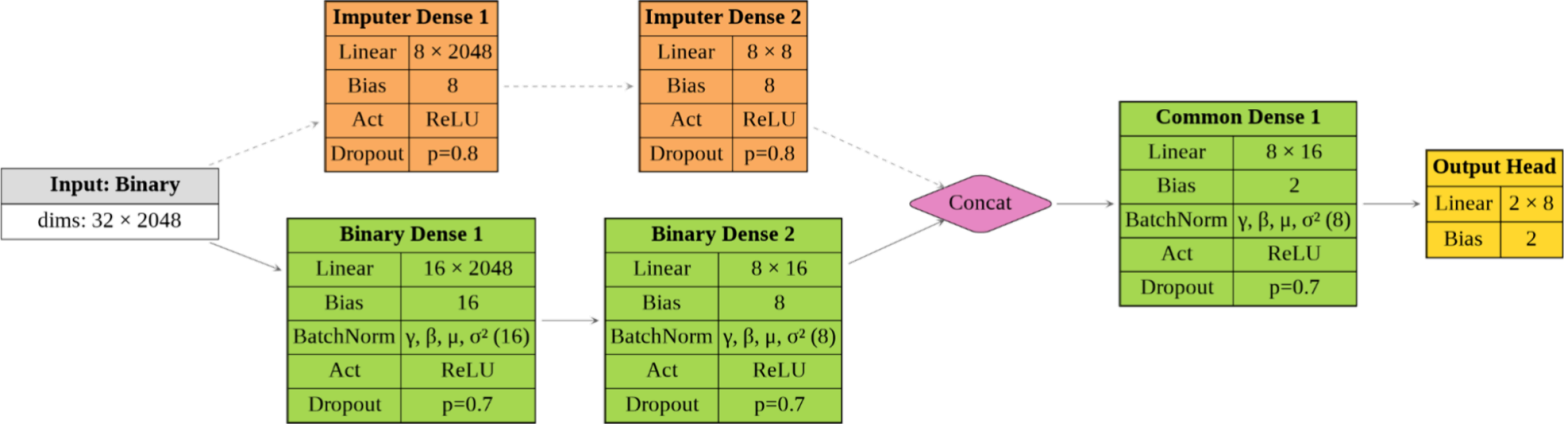
Schematic representation of the modified architecture with the imputer branch. The original temporal branch (see Figure 3) is replaced by an imputer module that reconstructs its latent representation directly from the binary input. The binary vector is processed by both the original binary branch (bottom) and the imputer branch (top right).

To assess whether the imputation-augmented model retains both its classification performance and confidence-aware behavior, we evaluated it using the same confidence thresholding strategy adopted for the baseline model. Specifically, we tested the model on data where the fingerprint input was augmented with a reconstructed version of the dynamical matrix, generated via a dedicated imputer trained to map fingerprints to the original dynamics. Table 3 summarizes classification performance across thresholds. Compared to the baseline, the imputation-augmented model exhibits a trade-off, achieving greater performance consistency at the cost of overall accuracy.

**Table 3:** Classification Performance of the Imputation-Augmented Model under Different Confidence Thresholds on Training and Test Sets

	threshold = 0.5		threshold = 0.8		threshold = 0.95	
metric	train	test	train	test	train	test
accuracy	100.00%	89.29%	100.00%	75.00%	90.58%	57.14%
F1 score	100.00%	89.16%	100.00%	83.97%	94.80%	71.67%
Cohen’s Kappa	1.00	0.79	1.00	0.59	0.83	0.40

At the default confidence threshold of 0.5, the imputation-augmented model demonstrated strong generalization capabilities, comparable to the original model. It achieved 89.29% accuracy on the test set, with an F1-score of 89.16% and a Cohen’s kappa of 0.79, indicating substantial agreement beyond chance. As shown in the test confusion matrix (Figure 8), all orthosteric samples were correctly classified, while 6 allosteric samples were misclassified as orthosteric. These results suggest that the model effectively leverages the reconstructed features to support robust and confident predictions.

**Figure 8:**
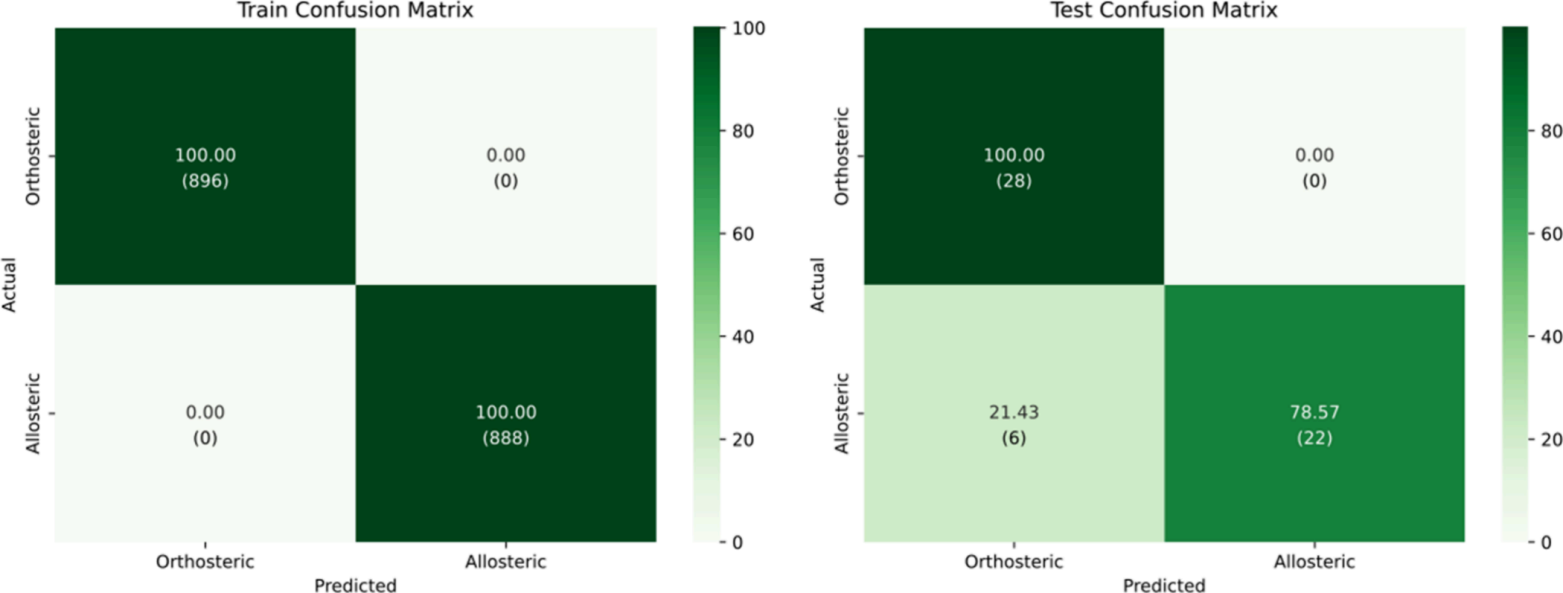
Confusion matrix for train and test sets using imputed dynamical features at confidence threshold = 0.5.

As the confidence threshold increased to 0.8, the model became more conservative in its predictions. Although test accuracy dropped to 75.00% and Cohen’s kappa fell to 0.59, the model maintained a strong F1-score of 83.97%, demonstrating a performance level comparable to the baseline. The confusion matrix in Figure 9further illustrates this trade-off: among the orthosteric samples, 22 were correctly classified and 6 were assigned to the uncertain class. For the allosteric class, 20 samples were correctly classified, 2 were misclassified as orthosteric, and 6 were labeled as uncertain. No samples were confidently assigned to the uncertain class, confirming that the model filtered out ambiguous cases rather than misclassifying them, thereby improving reliability at the cost of coverage.

**Figure 9:**
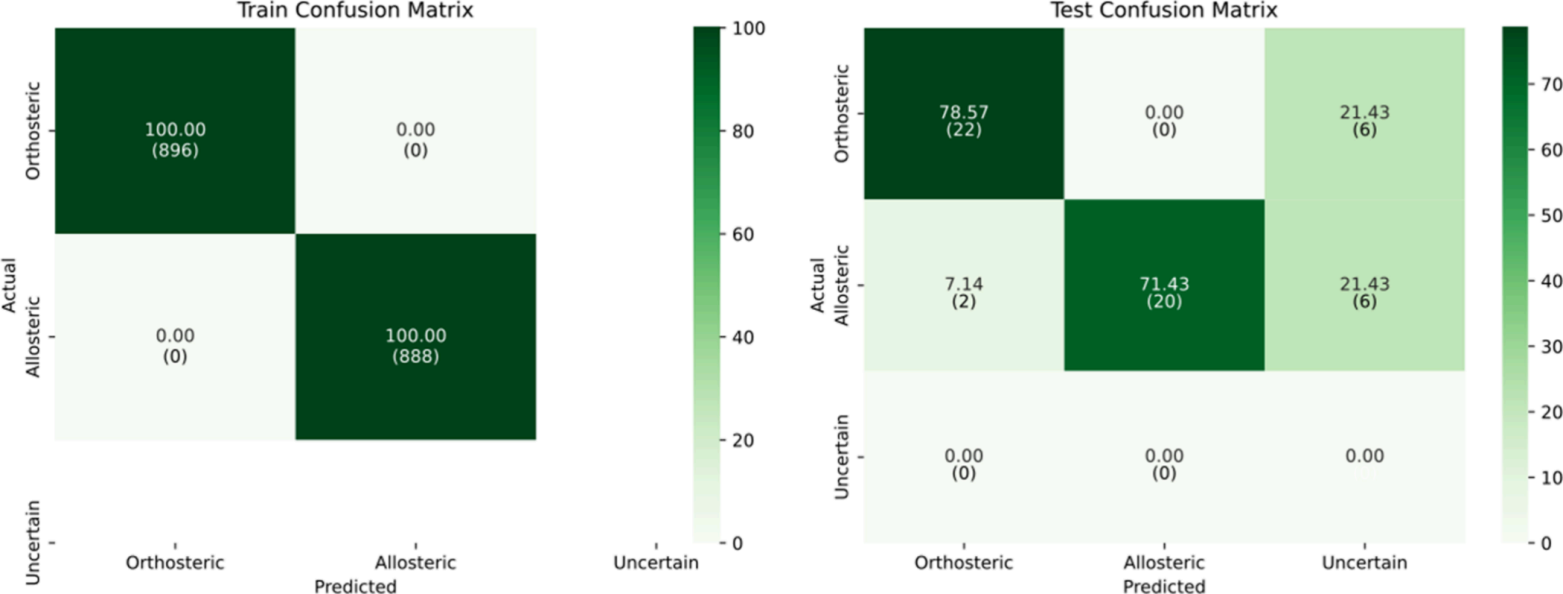
Confusion matrix for train and test sets using imputed dynamical features at confidence threshold = 0.8.

At the most conservative threshold of 0.95, the model exhibited a marked reduction in test performance. Test accuracy fell to 57.14%, with an F1-score of 71.67% and a Cohen’s kappa of 0.40. This performance degradation reflects the challenge of confidently predicting with reconstructed dynamics, as the model now assigned a larger portion of test samples to the uncertain class, as shown in Figure 10.

**Figure 10:**
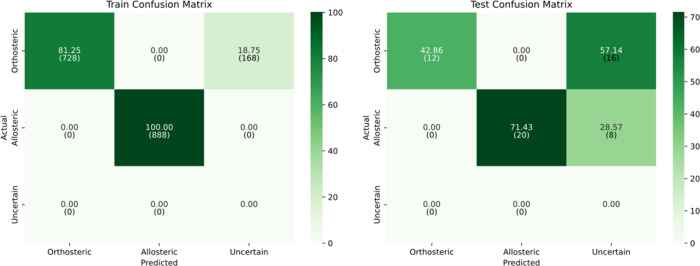
Confusion matrix for train and test sets using imputed dynamic features at confidence threshold = 0.95.

These results highlight a trade-off between prediction coverage and confidence in the imputed setting. While the imputed features supported high-confidence classification at lower thresholds, increasing the threshold led to a greater number of uncertain classifications and reduced overall accuracy. Still, the model preserved the desired behavior of avoiding false confident predictions. This demonstrates that, even in the absence of original dynamical information, the model can adaptively calibrate its predictions based on the confidence of its internal representations. Full tabulations of the per-ligand classification outcomes are reported in the Supporting Information (Tables S5–S7).

Given the promising results of the imputation-augmented model, we integrated the imputation branch into the original classifier architecture, leading to a model that is depicted in Figure 11. By combining these architectures, users can decide whether to use their own MD-derived data (if available) or to simply use the ligand’s fingerprint and impute the missing dynamical descriptors.

**Figure 11:**
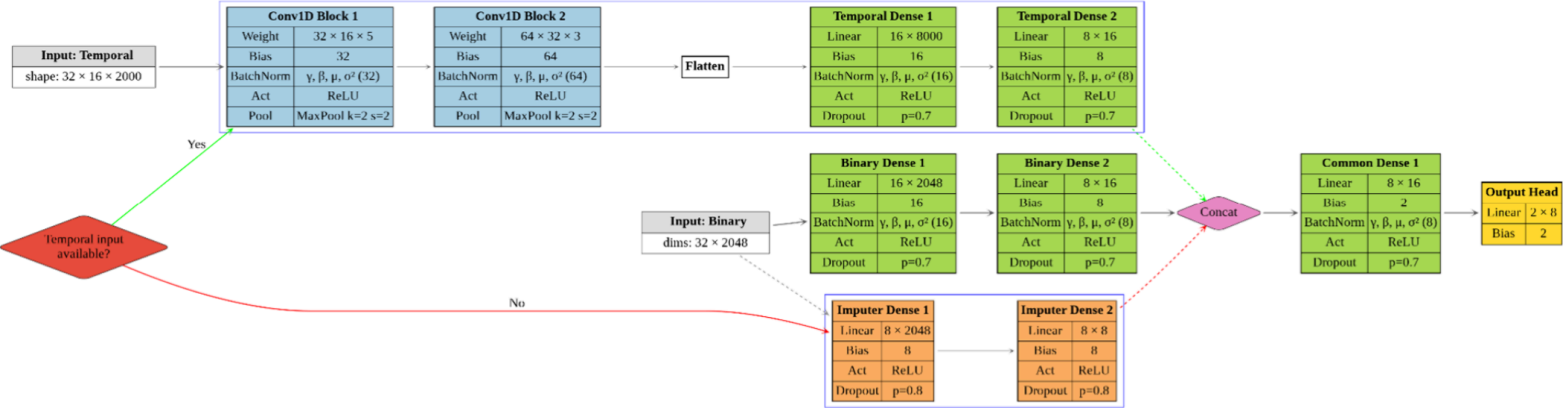
Schematic representation of the complete proposed deep learning model. The architecture integrates both temporal and binary inputs to perform classification. The temporal branch (top) processes time-series data using two convolutional blocks followed by dense layers. The binary branch (middle) directly processes binary feature vectors. An imputer branch (bottom) reconstructs the latent temporal representation from the binary input when temporal data is unavailable, using a dedicated reconstruction loss. The outputs of the temporal (or imputed) and binary branches are concatenated and passed to a common dense block, followed by a final classification head. Conditional routing allows the model to adapt to the presence or absence of temporal input at inference time.

## Discussion

In this work, we have developed a dual-modality deep learning model for the classification of ligand function on proteins in terms of orthosteric and allosteric regulation. The model is trained on both dynamics of the ligand-protein complexes and the chemical structure of the ligands, and represents a significant methodological advancement over previous studies.

First, we curated a data set of experimentally resolved ligand-protein structures that includes 73 different proteins belonging to the protein kinase family, for a total of 280 structures (with a 50:50 ratio of orthosteric and allosteric ligands). Kinases were selected as they represent key drug targets^48,49^ which have been widely investigated for lead development.^50,51^

This constitutes a significant enhancement and generalization of the data compared to previous studies, where only the Cyclin Dependent Kinase group (10 proteins) was considered, with the size of the data set consisting of only 38 structures. Furthermore, the dynamical data generated from a single MD replica was used as a complete matrix, encoding the entire simulation, and not only selected frames. By leveraging full dynamic evolution, we enable the model to learn from patterns that may span across different time scales, potentially improving its ability to discriminate between ligand functional classes through their actual impact on functional motions.

The second advance presented in this work is the architecture developed for the classification task. When investigating the distribution of data points belonging to each class, the embedding revealed substantial overlap between the two classes in latent space (Figure 2). This result suggests that linear or naive decision boundaries are unlikely to perform well and highlights the need for deep learning architectures capable of extracting subtle, nonlinear patterns from both the dynamical and structural representations.

Therefore, the proposed model consists of a temporal branch (CNN-based) which learns from the time series matrix that encodes protein motion, a binary branch (FCN-based) which learns from the molecular fingerprint that encodes ligand structure, and a common branch where the previous two merge together to predict the classification output.

In particular, each branch was tailored to maximize the learning of information encoded in the different types of inputs.

We evaluated the model’s output against three confidence thresholds: 0.5, 0.8, and 0.95. This mechanism allowed us to clearly separate high-confidence predictions from uncertain ones, thereby enhancing model interpretability and trustworthiness.

In this context, the adoption of a dynamic focal loss with entropy regularization proved especially beneficial. The dynamic focal loss gradually increases the focusing parameter γ during the initial training epochs, allowing the model to prioritize more challenging, low-confidence samples, resonating with.^52^ This is particularly well aligned with the decision strategy, as it explicitly pushes the model to improve its performance on the very predictions, most likely to fall into the uncertain category. The added entropy regularization further reinforces this behavior by penalizing overly ambiguous output distributions, encouraging the model to produce more decisive classifications.^28^ Together, these choices led to improved robustness and a clearer separation between confident and uncertain predictions.

Besides this first architecture, we trained a second model, in which we introduced an imputer mechanism to reconstruct the latent representation of the temporal branch directly from static ligand features. The goal for this second model was to address real-world constraints where molecular dynamics (MD) simulations may be unavailable or computationally impractical.

This approach is conceptually aligned with recent advances^53^ in deep learning-based imputation methods, particularly in omics domains, where latent representations are often inferred from partial or missing high-dimensional data. This conditional execution scheme enhances the model’s flexibility and applicability, especially in settings where the ligand binding site is unknown and performing multiple simulations would be prohibitively expensive. Importantly, this design preserves the architectural integrity of the original model while introducing a lightweight, plug-in solution for scenarios with incomplete input modalities. The feasibility and utility of such imputation strategies have gained increasing attention in molecular machine learning, particularly for tasks involving structure-based virtual screening or function prediction under resource constraints.^54,55^ Our results demonstrate the effectiveness of incorporating a confidence-based threshold to regulate model predictions and enable uncertainty-aware decision-making. At the default softmax threshold of 0.5, the model classified all samples confidently, achieving high accuracy (92.86%), F1-score (92.86%), and a strong Cohen’s kappa of 0.86, while the imputed model reached 89.29% accuracy, 89.16% F1-score with a slightly lower kappa of 0.79. This suggests that, when allowed to make predictions on all inputs, the model generalizes well and produces predictions with high reliability. Moreover, while the imputed features preserve meaningful signals, they introduce a degree of uncertainty that slightly compromises classification fidelity. However, relying solely on standard thresholds can obscure the reliability of individual predictions, especially in cases of overlapping class distributions or limited training data. By increasing the decision threshold to 0.8, the model became more selective – refusing to classify less confident samples and assigning them to an ″uncertain″ class. This trade-off led to a slight reduction in overall accuracy (85.71%) and F1-score (89.73%), but improved precision and interpretability by avoiding potentially misleading predictions. Notably, Cohen’s kappa remained relatively strong (0.74), indicating that the model still maintained meaningful predictive agreement despite abstaining from some classifications. At the same threshold of 0.8, the imputed model exhibited a more pronounced drop in performance, achieving 75.00% accuracy, 83.97% F1-score, and a kappa of 0.59. This larger decrease reflects the added uncertainty introduced by reconstructing the dynamic descriptors, which affects the model’s ability to confidently separate ambiguous samples. Nevertheless, the imputed model preserved the core benefit of the thresholding strategy – namely, filtering out low-confidence predictions to enhance reliability and interpretability.

At the highest threshold of 0.95, both models adopted a highly conservative stance. This resulted in a significant drop in test accuracy (69.64%) and Cohen’s kappa (0.52), while still preserving a decent F1-score (80.17%) on confidently predicted samples for the original model, whereas the imputed model saw further performance degradation, reaching 55.36% accuracy, 73.52% F1-score, and a kappa of 0.29. Although the models refrained from making many predictions, the strategy effectively filtered out low-confidence, ambiguous cases.

This cautious behavior can be highly beneficial in real-world applications, such as drug discovery or protein design, where incorrect confident predictions may lead to wasting experimental resources or false biological interpretations. The observed trend highlights a key trade-off: as confidence thresholds increase, the number of predictions decreases, but their reliability improves. Thus, the inclusion of an uncertainty-aware classification strategy empowers the model to prioritize reliable output over coverage, which can be especially valuable in domains involving noisy or heterogeneous data. This strategy aligns with the growing focus on selective classification and rejection learning in deep learning, where models are encouraged to abstain from uncertain decisions.^56^ Our implementation of confidence thresholds introduces a lightweight yet effective mechanism for this purpose, without requiring architectural changes or additional supervision. In practice, setting the threshold to 0.8 emerges as a favorable trade-off, preserving strong predictive performance while filtering out a small number of ambiguous cases. This behavior is especially valuable in experimental or clinical contexts, where interpretability and trust are critical.

Finally, to complement this uncertainty-aware framework and better understand the model’s internal reasoning, we performed a feature attribution analysis to quantify the contributions of each input modality – dynamical descriptors and molecular fingerprints – to the final predictions. Using Integrated Gradients (IG), we computed total and average attributions separately for the two input branches. The results revealed that the dynamical matrix input dominates overall predictive contribution, accounting for over 90% of the total attribution across both orthosteric and allosteric classes. However, when examining the average attribution per feature, the fingerprint input shows higher relative importance (∼ 62%), likely due to its lower dimensionality. This suggests that while dynamical features provide a broad and rich source of signal, their contribution is distributed across many features, whereas fingerprints offer more concentrated predictive cues. Importantly, the dominance of the dynamical descriptors also translates into improved reliability under stricter confidence thresholds. As shown in the Supporting Information (Table S1), a model relying solely on the binary fingerprint branch suffers substantial drops in accuracy and agreement when the threshold increases, highlighting its limited robustness. In contrast, the multimodal model, driven by the dynamical input, sustains much stronger performance, underscoring the importance of these features not only for predictive power but also for interpretability and stability in uncertainty-aware settings. The complementary roles of these inputs reinforce the strength of the multimodal design and point to possible improvements in interpretability and efficiency – such as dimensionality reduction to enhance the saliency of the dynamical descriptors. Together with the confidence-based thresholding mechanism, this feature importance analysis enhances our understanding of the model’s decision process and supports its application in high-stakes domains that demand both performance and explainability.

## Conclusions

In this work we have introduced a sophisticated dual-modality deep learning framework that accurately classifies ligand function as orthosteric or allosteric by integrating protein dynamics and ligand chemical structures. Our model represents a significant methodological advancement, built upon a large and diverse data set of protein kinases and uniquely leveraging complete molecular dynamics trajectories to capture the impact of ligands on the salient functionally oriented traits of protein motions. The architectures we devised effectively learn the complex, nonlinear patterns inherent to allosteric or orthosteric effects.

A key innovation of our study is the implementation of an uncertainty-aware classification strategy using confidence thresholds, which allows the model to refrain from generating ambiguous predictions. This significantly enhances the reliability and trustworthiness of its outputs, establishing a crucial trade-off between predictive coverage and precision that is vital for real-world applications like drug discovery. Importantly, we developed a novel imputation model that preserves high performance even without computationally expensive simulation data, greatly expanding its practical utility for virtual screening applications. Feature attribution analysis confirmed the synergistic value of the multimodal design, demonstrating that while dynamical features provide the dominant predictive signal, ligand fingerprints offer more concentrated and efficient cues. Collectively, this work delivers a robust, interpretable, and flexible tool that advances the functional classification of protein ligands, setting the stage for its use in complementing classical and novel approaches in the selection of ligands for the regulation of protein functions.

## Supplementary Material



## Notes

Computational details on training and validation loss; tables reporting on the performance of different control models (PDF)
