# Supplementary material for: MOLECULE: Molecular-dynamics and Optimized deep Learning for Entropy-regularized Classification and Uncertainty-aware Ligand Evaluation
Source: J Chem Theory Comput. 2025 Sep 11;21(18):9186–99. doi: 10.1021/acs.jctc.5c01140 (PMC12461829; doi:10.1021/acs.jctc.5c01140)
Supplement: Supplementary file 1 [file ct5c01140_si_001.pdf]

Supporting information for

MOLECULE: Molecular-dynamics and Optimized deep Learning for Entropy-regularized Classification and Uncertainty-aware Ligand Evaluation

Ivan Cucchi,[1,#] Elena Frasnetti,[2,#] Francesco Frigerio,[3] Fabrizio Cinquini,[4] Silvia Pavoni,[3] Luca F. Pavarino,[1,*] Giorgio Colombo[2,*]

1) Dipartimento di Matematica "F. Casorati", Università di Pavia, Via Ferrata 5, 27100 Pavia Italy
2) Dipartimento di Chimica, Università di Pavia, Via Taramelli 12, 27100 Pavia, Italy
3) Department of Physical Chemistry, R&D Eni SpA, via Maritano 27, 20097 San Donato Milanese (Mi), Italy
4) Upstream & Technical Services – TECS/STES – Eni Spa, via Emilia 1, 20097 San Donato Milanese (Mi), Italy

# These authors contributed equally to this manuscript

* Corresponding authors: g.colombo@unipv.it; luca.pavarino@unipv.it

- <u>Training and validation loss.</u>
  The model was trained over 200 epochs using the AdaBelief optimizer with dropout and weight regularization to prevent overfitting.
  Both training and validation losses decrease rapidly in the early epochs, indicating effective learning. After approximately 50 epochs, the validation loss stabilizes while the training loss continues to decline slowly, suggesting good generalization. Early stopping was not triggered, as the validation loss remained relatively stable with minor fluctuations throughout the training.

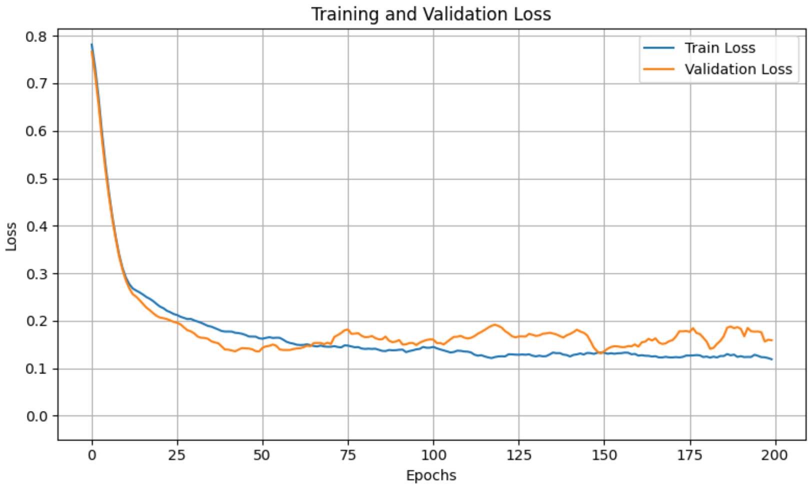

Figure S1. Training and validation loss curves.

- <u>Binary-only model's performance of the classification at different confidence thresholds.</u> While performance is reasonable at the standard 0.5 threshold (82.14% test accuracy, Cohen's kappa = 0.64), it deteriorates sharply at higher thresholds, with both accuracy and agreement dropping below 40% on the test set. These results highlight the limited reliability of the fingerprint-only branch and underscore the importance of the dynamical descriptors in ensuring robust and interpretable predictions under stricter confidence criteria.

	**Threshold = 0.5**		**Threshold = 0.8**		**Threshold = 0.95**	
**Metric**	**Train**	**Test**	**Train**	**Test**	**Train**	**Test**
Accuracy	99.55%	82.14%	49.78%	32.14%	49.33%	32.14%
F1 score	99.55%	81.55%	50.00%	39.13%	49.77%	39.13%
Cohen's Kappa	0.99	0.64	0.33	0.19	0.33	0.19

Table S1. Classification performance of the binary-only model under different confidence thresholds on training and test sets.

- <u>Results of the classification task at different confidence thresholds.</u>
  The following tables summarize the classification output for each ligand.
  Predicted classes are obtained from the model using different confidence thresholds; in particular, predictions below the chosen threshold are labeled as Uncertain.
  For ligands with multiple predictions, all predicted classes and their correctness are shown.
  ✔ for correct prediction, ✘ for incorrect prediction and ? for uncertain prediction.

PDB code	True Class	Predicted Class(es)	Check(s)
1m17	Orthosteric	Orthosteric	✔
1unl	Orthosteric	Orthosteric	✔
2b54	Orthosteric	Orthosteric	✔
3aox	Orthosteric	Orthosteric	✔
3d7z	Orthosteric	Orthosteric	✔
3g0e	Orthosteric	Orthosteric	✔
3lw0_2	Allosteric	Allosteric	✔
3py0	Orthosteric	Orthosteric	✔
3pyy	Allosteric	Allosteric	✔
4a07_1	Allosteric	Allosteric	✔
4a07_2	Allosteric	Allosteric	✔
4e6a	Allosteric	Allosteric	✔
4mne_1	Allosteric	Allosteric	✔
4qmm	Orthosteric	Orthosteric	✔
4zsl	Allosteric	Orthosteric	✘
5ack	Allosteric	Allosteric	✔
5n64	Allosteric	Allosteric	✔
5orp	Allosteric	Allosteric	✔
5ose	Allosteric	Allosteric	✔
5otp	Allosteric	Allosteric	✔
5tr6	Orthosteric	Orthosteric	✔
5ut4	Orthosteric	Orthosteric	✔
6bbv	Orthosteric	Orthosteric	✔
6vnh	Orthosteric	Orthosteric	✔
7ju5	Orthosteric	Orthosteric	✔
7t4v	Allosteric	Allosteric	✔
7vdr	Orthosteric	Allosteric	✘
7zyk	Allosteric	Allosteric	✔

Table S2. True and predicted binding site labels for each ligand in the test set at confidence threshold = **0.5**.

PDB code	True Class	Predicted Class(es)	Check(s)
1m17	Orthosteric	Orthosteric	✔
1unl	Orthosteric	Orthosteric	✔
2b54	Orthosteric	Orthosteric	✔
3aox	Orthosteric	Orthosteric	✔
3d7z	Orthosteric	Orthosteric	✔
3g0e	Orthosteric	Orthosteric	✔
3lw0_2	Allosteric	Allosteric	✔
3py0	Orthosteric	Orthosteric	✔
3pyy	Allosteric	Uncertain	❓
4a07_1	Allosteric	Allosteric	✔
4a07_2	Allosteric	Allosteric	✔
4e6a	Allosteric	Allosteric	✔
4mne_1	Allosteric	Allosteric	✔
4qmm	Orthosteric	Uncertain	❓
4zsl	Allosteric	Orthosteric	✘
5ack	Allosteric	Allosteric	✔
5n64	Allosteric	Allosteric	✔
5orp	Allosteric	Allosteric	✔
5ose	Allosteric	Allosteric	✔
5otp	Allosteric	Allosteric	✔
5tr6	Orthosteric	Orthosteric	✔
5ut4	Orthosteric	Orthosteric	✔
6bbv	Orthosteric	Orthosteric	✔
6vnh	Orthosteric	Orthosteric	✔
7ju5	Orthosteric	Orthosteric	✔
7t4v	Allosteric	Allosteric	✔
7vdr	Orthosteric	Uncertain / Allosteric	❓/ ✘
7zyk	Allosteric	Allosteric	✔

Table S3. True and predicted binding site labels for each ligand in the test set at confidence threshold = **0.8.**

PDB code	True Class	Predicted Class(es)	Check(s)
1m17	Orthosteric	Orthosteric	✓
1unl	Orthosteric	Uncertain	?
2b54	Orthosteric	Orthosteric / Uncertain	✓ / ?
3aox	Orthosteric	Orthosteric	✓
3d7z	Orthosteric	Orthosteric	✓
3g0e	Orthosteric	Uncertain / Orthosteric	? / ✓
3lw0_2	Allosteric	Allosteric	✓
3py0	Orthosteric	Uncertain / Orthosteric	? / ✓
3pyy	Allosteric	Uncertain	?
4a07_1	Allosteric	Allosteric	✓
4a07_2	Allosteric	Allosteric	✓
4e6a	Allosteric	Allosteric	✓
4mne_1	Allosteric	Allosteric	✓
4qmm	Orthosteric	Uncertain	?
4zsl	Allosteric	Orthosteric	✗
5ack	Allosteric	Allosteric	✓
5n64	Allosteric	Allosteric	✓
5orp	Allosteric	Uncertain	?
5ose	Allosteric	Allosteric	✓
5otp	Allosteric	Allosteric	✓
5tr6	Orthosteric	Orthosteric	✓
5ut4	Orthosteric	Orthosteric / Uncertain	✓ / ?
6bbv	Orthosteric	Orthosteric / Uncertain	✓ / ?
6vnh	Orthosteric	Orthosteric	✓
7ju5	Orthosteric	Orthosteric	✓
7t4v	Allosteric	Allosteric	✓
7vdr	Orthosteric	Uncertain	?
7zyk	Allosteric	Allosteric	✓

Table S4. True and predicted binding site labels for each ligand in the test set at confidence threshold = **0.95**.

- <u>Results of the classification task with imputed data at different confidence thresholds.</u>
  The following tables summarize the classification output for each ligand.

Predicted classes are obtained from the model augmented with imputed dynamical features using different confidence thresholds; in particular, predictions below the chosen threshold are labeled as Uncertain.
For ligands with multiple predictions, all predicted classes and their correctness are shown.
✔ for correct prediction, ✗ for incorrect prediction and ? for uncertain prediction.

PDB code	True Class	Predicted Class(es)	Check(s)
1m17	Orthosteric	Orthosteric	✔
1unl	Orthosteric	Orthosteric	✔
2b54	Orthosteric	Orthosteric	✔
3aox	Orthosteric	Orthosteric	✔
3d7z	Orthosteric	Orthosteric	✔
3g0e	Orthosteric	Orthosteric	✔
3lw0_2	Allosteric	Allosteric	✔
3py0	Orthosteric	Orthosteric	✔
3pyy	Allosteric	Allosteric	✔
4a07_1	Allosteric	Allosteric	✔
4a07_2	Allosteric	Allosteric	✔
4e6a	Allosteric	Allosteric	✔
4mne_1	Allosteric	Allosteric	✔
4qmm	Orthosteric	Orthosteric	✔
4zsl	Allosteric	Orthosteric	✗
5ack	Allosteric	Allosteric	✔
5n64	Allosteric	Allosteric	✔
5orp	Allosteric	Orthosteric	✗
5ose	Allosteric	Orthosteric	✗
5otp	Allosteric	Allosteric	✔
5tr6	Orthosteric	Orthosteric	✔
5ut4	Orthosteric	Orthosteric	✔
6bbv	Orthosteric	Orthosteric	✔
6vnh	Orthosteric	Orthosteric	✔
7ju5	Orthosteric	Orthosteric	✔
7t4v	Allosteric	Allosteric	✔
7vdr	Orthosteric	Orthosteric	✔
7zyk	Allosteric	Allosteric	✔

Table S5. True and predicted binding site labels for each ligand in the test set using the imputed dynamical features at confidence threshold = **0.5**.

PDB code	True Class	Predicted Class(es)	Check(s)

1m17	Orthosteric	Orthosteric	✓
1unl	Orthosteric	Orthosteric	✓
2b54	Orthosteric	Uncertain	?
3aox	Orthosteric	Orthosteric	✓
3d7z	Orthosteric	Uncertain	?
3g0e	Orthosteric	Uncertain	?
3lw0_2	Allosteric	Allosteric	✓
3py0	Orthosteric	Orthosteric	✓
3pyy	Allosteric	Uncertain	?
4a07_1	Allosteric	Allosteric	✓
4a07_2	Allosteric	Allosteric	✓
4e6a	Allosteric	Allosteric	✓
4mne_1	Allosteric	Allosteric	✓
4qmm	Orthosteric	Orthosteric	✓
4zsl	Allosteric	Orthosteric	✗
5ack	Allosteric	Allosteric	✓
5n64	Allosteric	Allosteric	✓
5orp	Allosteric	Uncertain	?
5ose	Allosteric	Uncertain	?
5otp	Allosteric	Allosteric	✓
5tr6	Orthosteric	Orthosteric	✓
5ut4	Orthosteric	Orthosteric	✓
6bbv	Orthosteric	Orthosteric	✓
6vnh	Orthosteric	Orthosteric	✓
7ju5	Orthosteric	Orthosteric	✓
7t4v	Allosteric	Allosteric	✓
7vdr	Orthosteric	Orthosteric	✓
7zyk	Allosteric	Allosteric	✓

Table S6. True and predicted binding site labels for each ligand in the test set using the imputed dynamical features at confidence threshold = **0.8**.

PDB code	True Class	Predicted Class(es)	Check(s)
1m17	Orthosteric	Uncertain	?
1unl	Orthosteric	Orthosteric	✓
2b54	Orthosteric	Uncertain	?

3aox	Orthosteric	Uncertain	❓
3d7z	Orthosteric	Uncertain	❓
3g0e	Orthosteric	Uncertain	❓
3lw0_2	Allosteric	Allosteric	✔
3py0	Orthosteric	Uncertain	❓
3pyy	Allosteric	Uncertain	❓
4a07_1	Allosteric	Allosteric	✔
4a07_2	Allosteric	Allosteric	✔
4e6a	Allosteric	Allosteric	✔
4mne_1	Allosteric	Allosteric	✔
4qmm	Orthosteric	Uncertain	❓
4zsl	Allosteric	Uncertain	❓
5ack	Allosteric	Allosteric	✔
5n64	Allosteric	Allosteric	✔
5orp	Allosteric	Uncertain	❓
5ose	Allosteric	Uncertain	❓
5otp	Allosteric	Allosteric	✔
5tr6	Orthosteric	Orthosteric	✔
5ut4	Orthosteric	Orthosteric	✔
6bbv	Orthosteric	Orthosteric	✔
6vnh	Orthosteric	Orthosteric	✔
7ju5	Orthosteric	Orthosteric	✔
7t4v	Allosteric	Allosteric	✔
7vdr	Orthosteric	Uncertain	❓
7zyk	Allosteric	Allosteric	✔

Table S7. True and predicted binding site labels for each ligand in the test set using the imputed dynamical features at confidence threshold = **0.95**.
